# Nanoluciferase Reporter Mycobacteriophage for Sensitive and Rapid Detection of Mycobacterium tuberculosis Drug Susceptibility

**DOI:** 10.1128/JB.00411-20

**Published:** 2020-10-22

**Authors:** Paras Jain, Spencer Garing, Deepshikha Verma, Rajagopalan Saranathan, Nicholas Clute-Reinig, Jacob Gadwa, Chelsea Peterson, Gleda Hermansky, Anna Astashkina Fernandez, Emmanuel Asare, Torin R. Weisbrod, Ethan Spencer, Claire V. Mulholland, Michael Berney, David Bell, Kevin P. Nichols, Anne-Laure M. Le Ny, Diane Ordway, William R. Jacobs, Akos Somoskovi, Kyle J. Minch

**Affiliations:** a Intellectual Ventures Laboratory, Bellevue, Washington, USA; b Department of Microbiology and Immunology, Albert Einstein College of Medicine, Bronx, New York, USA; c Trudeau Institute, Saranac Lake, New York, USA; d Mycobacteria Research Laboratories, Department of Microbiology, Immunology and Pathology, Colorado State University, Fort Collins, Colorado, USA; e Department of Pathology, Albert Einstein College of Medicine and Montefiore Medical Center, Bronx, New York, USA; f Intellectual Ventures’ Global Good Fund, Bellevue, Washington, USA; g Department of Molecular Genetics, Albert Einstein College of Medicine, Bronx, New York, USA; Brigham and Women's Hospital/Harvard Medical School

**Keywords:** *Mycobacterium tuberculosis*, bacteriophages, drug screening, drug susceptibility testing, nanoluciferase, phage

## Abstract

Phenotypic testing for drug susceptibility of Mycobacterium tuberculosis is critical to basic research and managing the evolving problem of antimicrobial resistance in tuberculosis management, but it remains a specialized technique to which access is severely limited. Here, we report on the development and validation of an improved phage-mediated detection system for M. tuberculosis. We incorporated a nanoluciferase (Nluc) reporter gene cassette into the TM4 mycobacteriophage genome to create phage TM4-nluc. We assessed the performance of this reporter phage in the context of cellular limit of detection and drug susceptibility testing using multiple biosafety level 2 drug-sensitive and -resistant auxotrophs as well as virulent M. tuberculosis strains. For both limit of detection and drug susceptibility testing, we developed a standardized method consisting of a 96-hour cell preculture followed by a 72-hour experimental window for M. tuberculosis detection with or without antibiotic exposure. The cellular limit of detection of M. tuberculosis in a 96-well plate batch culture was ≤10^2^ CFU. Consistent with other phenotypic methods for drug susceptibility testing, we found TM4-nluc to be compatible with antibiotics representing multiple classes and mechanisms of action, including inhibition of core central dogma functions, cell wall homeostasis, metabolic inhibitors, compounds currently in clinical trials (SQ109 and Q203), and susceptibility testing for bedaquiline, pretomanid, and linezolid (components of the BPaL regimen for the treatment of multi- and extensively drug-resistant tuberculosis). Using the same method, we accurately identified rifampin-resistant and multidrug-resistant M. tuberculosis strains.

**IMPORTANCE** Mycobacterium tuberculosis, the causative agent of tuberculosis disease, remains a public health crisis on a global scale, and development of new interventions and identification of drug resistance are pillars in the World Health Organization End TB Strategy. Leveraging the tractability of the TM4 mycobacteriophage and the sensitivity of the nanoluciferase reporter enzyme, the present work describes an evolution of phage-mediated detection and drug susceptibility testing of M. tuberculosis, adding a valuable tool in drug discovery and basic biology research. With additional validation, this system may play a role as a quantitative phenotypic reference method and complement to genotypic methods for diagnosis and antibiotic susceptibility testing.

## Abstract (precis)

Mycobacterium tuberculosis, the causative agent of tuberculosis disease, remains a public health crisis on a global scale, and development of new interventions and identification of drug resistance are pillars in the World Health Organization End TB Strategy. Leveraging the tractability of the TM4 mycobacteriophage and the sensitivity of the nanoluciferase reporter enzyme, the present work describes an evolution of phage-mediated detection and drug susceptibility testing of M. tuberculosis, adding a valuable tool in drug discovery and basic biology research. With additional validation, this system may play a role as a quantitative phenotypic reference method and complement to genotypic methods for diagnosis and antibiotic susceptibility testing.

## INTRODUCTION

Tuberculosis (TB), caused by Mycobacterium tuberculosis, is a disease with an effective antibiotic therapy consisting of a combination of 4 antibiotics taken for 6 months; however, the emergence of strains with fewer options for curative therapy (multi-, extensively, and totally drug-resistant strains [MDR-TB, XDR-TB, and TDR-TB, respectively]) presents a public health threat and underscores the need for advancements in tools to facilitate new drug development as well as to diagnose and effectively treat this disease (1, 2). Accordingly, intensified research on interventions and identification of drug resistance to guide appropriate therapy are pillars in the WHO End TB Strategy for disease elimination (1, 3).

For drug susceptibility testing (DST), methods are broadly divided between phenotypic and genotypic approaches. Genotypic assays are attractive due to their rapid turnaround time and capacity to probe multiple predetermined loci in a single reaction (4–6). While data considering diverse antibiotics and multiple resistance loci in M. tuberculosis are accumulating from next-generation sequencing efforts (7), genotypic DST is most effective for drugs with well-defined resistance mutations, as in the GeneXpert M. tuberculosis/RIF Ultra test (8). Identification of MDR-TB and XDR-TB is more complex, in part due to our incomplete understanding of resistance mutations to all the 10-plus antibiotics that can be used in these treatment regimens and the potential for new mutations to arise (9). Recent results with a streamlined MDR/XDR treatment regimen (bedaquiline, pretomanid, and linezolid [BPaL]) demonstrate the efficacy of simplified therapy for drug-resistant tuberculosis (10, 11); however, resistance mutations are still being identified, and phenotypic testing is necessary for initial identification of these (12). Thus, the diversity of antibiotics used to treat TB, and the lack of correlation between a genotype and the resulting phenotypic resistance, call for complementary approaches in both viability monitoring to identify genotype-to-phenotype correlations in basic research, as well as for clinical reference.

For decades, phenotypic growth-based assays have been a reference method for M. tuberculosis detection and quantitative DST and are critical for identifying resistance mutations that can be probed in rapid genotypic tests (13). These approaches are appealing in that they provide a readout of viability and/or drug susceptibility based on detection of expanding bacterial populations and are generally agnostic to antibiotic mechanism or prospective knowledge of underlying resistance mutations. For drug-susceptible cells responding to antibiotic treatments, metabolic homeostasis is compromised, whereas the drug-resistant cells exposed to antibiotic treatment maintain homeostasis and division. The potency of a given compound against M. tuberculosis is subsequently quantified by measuring whole-cell responses across a range of treatment concentrations, which correspondingly allows for interpretation of the level of resistance or susceptibility that an M. tuberculosis strain exhibits against an antibiotic.

Two common measures of antibiotic potency/M. tuberculosis susceptibility are minimal bactericidal concentration (MBC) and the MIC. MBC assays define the ability of a test compound to kill M. tuberculosis, whereas MIC values describe the concentration at which an antibiotic inhibits growth to a defined level (typically reported at 95 or 99%) compared to untreated control cells (14, 15). While the MBC and MIC are both variations on the theme of DST and broad “viability monitoring” because the MBC requires distinct exposure and recovery/regrowth periods, they are a preferred research tool for identification and characterization of anti-TB agents (15). MIC data have been widely used by WHO to define the critical concentrations, epidemiological cutoff values (ECOFFs), and clinical breakpoint of anti-TB agents to guide individual clinical decisions in patient treatment (16).

Multiple approaches, including the agar proportion method, Mycobacterial Growth Indicator Tube (MGIT) system (17), microscopic observation of drug susceptibility (MODS) (18), and Sensititre broth microdilution assays (19), can be used to determine MIC and MBC values; however, the time to result is dependent on the starting inoculum, and, in the case of M. tuberculosis with a doubling time of >16 hours, a positive culture result requires days to weeks of incubation. Recognizing the need for accurate drug susceptibility testing and as a complement to the increasing potential of decentralized DST by genotypic methods, different strategies have been developed to decrease the time to result of replication-based phenotypic assays (20, 21). In addition to these, phenotypic methods that rely on increasingly sensitive detection of proxy (nonreplication) signals while still maintaining the genotype-agnostic features of a growth-based viability detection assay are a parallel path for understanding the response of M. tuberculosis to a test condition (22).

Reporter mycobacteriophage systems represent a method for deriving phenotypic data in a replication-independent system, maintaining high sensitivity while reducing the time to result attendant with growth-based methods (23). Mycobacteriophages are viruses specific to mycobacteria and require viable M. tuberculosis for their propagation. In reporter systems, the virus attaches to cell surface and injects its genetic material into the susceptible host, which then transcribes and translates the foreign DNA, including the reporter gene (24). Inhibition of M. tuberculosis homeostasis by various antibiotics inhibits the expression of the reporter gene and compromises signal, thus providing a readout analogous to other growth/culture-based phenotypic assays but in a faster time frame. The first reporter mycobacteriophage was derived from mycobacteriophage TM4 engineered to encode a firefly luciferase (ffLuc) gene cassette (25). The performance of this system was subsequently improved through methods development (26) and phage modifications (27, 28), enabling studies using phage for M. tuberculosis detection and DST from isolates and clinical specimens (29, 30). Additional phage modifications, including the introduction of a stronger constitutive promoter and use of reporter cassettes encoding one or more fluorescent proteins (fluorophages), enabled differentiation of antibiotic tolerance versus resistance in a mixed bacterial population, measurement of bactericidal activity of antibiotics against M. tuberculosis *in vitro* and in *ex vivo* sputum samples from TB patients, and DST assays directly on sputum samples from TB patients identifying rifampin heteroresistance (31–35). The combined conclusions of these efforts indicate that it is possible to use the TM4 mycobacteriophage encoding different reporter gene cassettes for DST and to measure bactericidal activity of anti-M. tuberculosis agents. The existing reporter phages, however, require sophisticated infrastructure to measure signal (fluorescence microscopy or flow cytometry), or, in the case of luciferase reporters to date, maximum sensitivity or signal stability is compromised due to intrinsic properties of the luciferase implemented (31, 32, 34, 36).

In an effort to improve the performance of previously described TM4 systems, in the present work, we created a TM4 phage vector that delivers a gene cassette of the nanoluciferase (Nluc) reporter enzyme (36; https://www.promega.com/resources/technologies/nanoluc-luciferase-enzyme/), which we term “TM4-nluc.” We tested this TM4-nluc phage on auxotrophic and virulent clinical M. tuberculosis strains, assessing cellular limit of detection and compatibility with antibiotic susceptibility testing from a range of antibiotic classes/mechanisms of action. We found that following a preculture period, using TM4-nluc, the cellular limit of detection was on the order of high tens/low hundreds of M. tuberculosis cells and that in a 72-hour combined drug treatment/phage infection, we could identify drug susceptibility consistent with WHO-endorsed treatment concentrations to first-line antibiotics (rifampin, isoniazid, and ethambutol), a suite of second-line antibiotics, including all components of the BPaL regimen (bedaquiline, pretomanid, and linezolid), as well as compounds currently in trials to assess efficacy as TB therapeutics (SQ109 and Q203). In summary, we report here on the development, and methods to use, of an improved detection assay that is capable of identifying very low numbers of M. tuberculosis cells in batch culture, as well as drug susceptibility testing for antibiotics with mechanisms of action ranging from core “central dogma” functions (transcription, translation, and DNA replication) to metabolic and cell wall homeostasis, all in a 72-hour drug exposure-to-result time period.

## RESULTS

### Generation of TM4-nluc phage and methods for its use.

Similar to firefly luciferase (ffLuc), Nluc allows for detection of batch signal from cells in well plate format; however, compared to ffLuc, Nluc generates a >10-fold-brighter signal on a molecule-per-molecule basis and utilizes a furimazine substrate that does not require ATP for light generation (37). Based on these reported performance characteristics of Nluc, we modified the parent phAE159 mycobacteriophage genome by cloning an M. tuberculosis codon-optimized Nluc gene (*nluc*_mtb_) (see Fig. S1 in the supplemental material) downstream of the constitutively active L5 promoter (PL_L5_:*nluc*_mtb_) (31, 38). We term this new phage reagent “TM4-nluc.”

Following generation of the base TM4 reporter construct, we proceeded with propagation of the phage stock as described previously (see Materials and Methods and reference 31). The Mycobacterium smegmatis plate harvest method yielded crude phage stocks in MP buffer in the range of 109 to 1,010 PFU/ml; however, due to the constitutively active nature of the promoter controlling reporter gene expression and the stability of Nluc, we observed high levels of active/functional Nluc as a by-product in phage preps, which, if left intact, diminished signal/noise ratios and compromised sensitivity in downstream experiments and assays. We assessed multiple methods for purifying contaminating Nluc away from TM4-nluc phage and ultimately identified two protocols (see Materials and Methods) that provided an optimal balance between phage purity and viability. Whether performing purification by ultracentrifugation or a multistep protocol incorporating centrifugation, anion-exchange chromatography, and hydrophobic interaction chromatography in the purified TM4-nluc phage preps, the background Nluc activity was reduced by >3 log. When normalized to PFU, there was <1-log phage loss between the initial and purified TM4-nluc phage preps.

**FIG 1:**
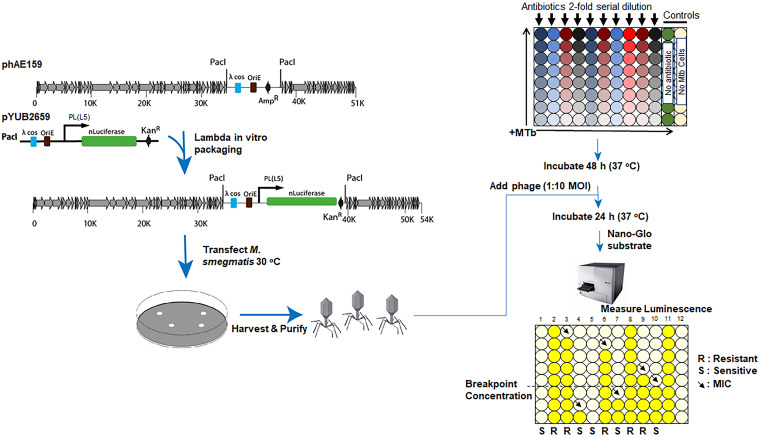
Schematic overview of phage reporter construct and method. TM4-nluc derives from modifying the pYUB2659 vector with a nanoluciferase gene cassette codon optimized for expression in M. tuberculosis (MTb). Following methods in reference 60, the reporter gene cassette was inserted into the phAE159 (TM4) backbone. Following lambda phage *in vitro* packaging, the reporter phasmid was transfected into M. smegmatis for phage amplification and expansion. TM4-nluc was sequentially purified (see Materials and Methods) prior to being deployed in M. tuberculosis detection or DST assays. Shown here, DST assays were conducted on uniform M. tuberculosis CFU inputs in each well with a 2-fold serial dilution of antibiotics (95 μl) proceeding down columns of a 96-well microtiter plate. After a 48-hour incubation period at 37°C in which M. tuberculosis cells were exposed to antibiotics (or control conditions), 5 μl phage was added to the appropriate wells, and the plate was returned to 37°C for a 24-hour infection/incubation. Following the 24-hour infection, 50 μl Nano-Glo substrate (prepared to manufacturer instructions) was added, and plates were read immediately on a BioTek Synergy H1 plate reader (refer to Materials and Methods). In the schematic depicted here, column 1 is populated by complete drug-sensitive M. tuberculosis cells (S); the drug treatment inhibits nanoluciferase production corresponding to a decrease in light signal generation. Column 2 is populated by complete drug-resistant cells (R), with maximum nanoluciferase production and light generation independent of the antibiotic concentration. Reading from top to bottom, black arrows indicate the drug dilution where antibiotic exposure falls below an inhibitory threshold above which nanoluciferase light production is inhibited and below which nanoluciferase light production approaches the untreated controls. Control wells include cells only, no phage; cells plus phage, no antibiotic; phage only, no cells; and empty medium wells.

With purified TM4-nluc in hand, we developed an experimental system to assess this new reporter reagent for viability of M. tuberculosis in the context of (i) sensitivity/cellular limit of detection and (ii) drug susceptibility/resistance testing. The workflow is presented schematically in Fig. 1 and Fig. S2 and described in greater detail in the subsequent text.

During the course of development, we identified critical components for maximizing assay performance. We observed that a “preculture” period maximizes signal/noise ratios of the viability assay. Accordingly, in our *in vitro* system, M. tuberculosis cells (virulent or auxotroph) were inoculated to a low target cell density and incubated at 37°C for 72 hours (∼3 to 4 doublings for M. tuberculosis strains reported here). Although the exact mechanism is unclear, the presence of detergents commonly used in M. tuberculosis culture strongly inhibits phage infection and removal of detergent, and subsequent incubation in detergent-free medium was critical in maximizing signal/noise. Following equilibration in detergent-free medium, the M. tuberculosis cells were used in detection/viability monitoring assays in 96-well plate format (with or without antibiotic exposure). Independent of the treatment parameters of an experiment, in this system, TM4-nluc phage was added at a multiplicity of infection (MOI) >1 and <1,000 and incubated for 24 hours at 37°C. At the conclusion of the 24-hour infection/incubation, a fixed amount of Nluc substrate, Nano-Glo, was added to the wells, and a luminescent signal was measured by standard plate reader.

Whether conducting experiments for strict M. tuberculosis detection or DST, this TM4 system is a reporter of metabolic activity in the cells. In the event that viable cells with intact metabolic homeostasis are present, the TM4-nluc phage infect M. tuberculosis, and the PL_L5_:*nluc*_mtb_ expression cassette is transcribed and translated, resulting in reporter enzyme production. Due to the temperature-sensitive nature of the phage, this incubation at 37°C should not lead to lysis of M. tuberculosis cells, and the majority of enzyme remains intracellular; however, in experiments that implemented filter plates and vacuum washing of infected cells to reduce further background Nluc, we observed a reduction in overall signal from washed assay compared to the no-wash assay format (Fig. S3). This result suggests that enzyme exists in the extracellular milieu due to either protein translocation or cell lysis and underscores the importance of phage production/purification to optimize signal/noise in a one-pot assay.

### Phage-mediated M. tuberculosis limit of detection is ≤10^2^ CFU, and signal improves with increased culture acclimation time.

**FIG 2:**
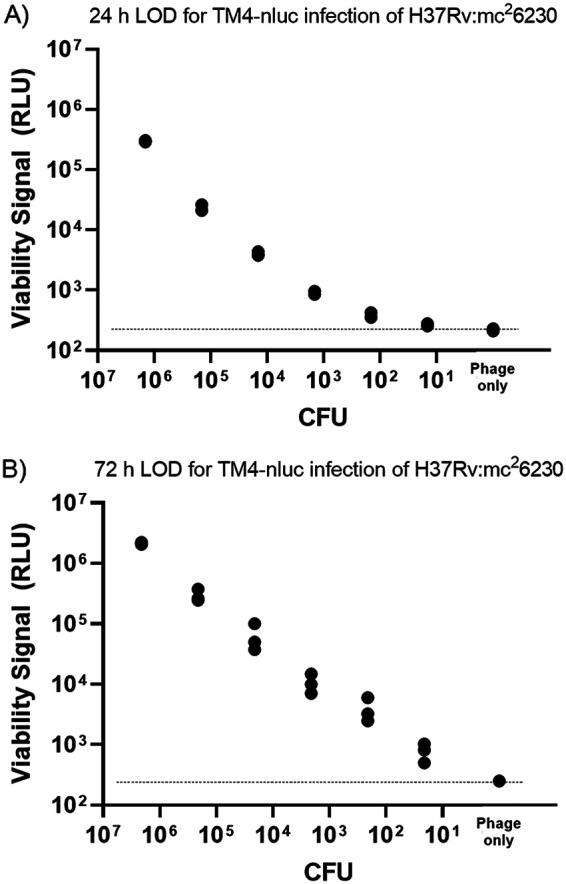
Phage-mediated M. tuberculosis limit of detection is ≤10^2^ CFU, and signal improves with increased culture acclimation time. After a preculture period in rich growth medium with detergent, M. tuberculosis cells were washed and resuspended in rich growth medium without detergent and inoculated in 10-fold serial dilution into 96-well microtiter plates. Phage was added immediately after transition to detergent-free medium to determine the 24-h LOD (A) or given a 48-hour acclimation period in the detergent-free growth medium followed by phage addition and 24 h of incubation to determine the 72-h LOD (B). Dashed lines indicate the noise floor as determined by phage-only background RLU (average of RLU from phage-only wells; *n* = 3). In both cases, the cellular limit of detection is in the range of low 10^2^ CFU/well.

Using the general method described above as a template, we assessed the cellular limit of detection (LOD) using TM4-nluc. In these experiments, cells were precultured as described above and in Materials and Methods and diluted in 10-fold serial steps. For these experiments, we investigated if an extended acclimation period in the plate (after transfer to detergent-free medium) affected the apparent relative luminescent unit (RLU) signal cellular LOD of the system. Accordingly, cells were inoculated in the plate and either immediately infected with TM4-nluc and incubated overnight at 37°C (Fig. 2A) or inoculated into the 96-well plate and allowed to equilibrate in the plate with detergent-free media for 48 hours prior to infection with TM4-nluc and overnight incubation (Fig. 2B). Immediately prior to phage addition, an aliquot of cells was removed, diluted in media containing detergent, and plated on 7H10 plates for CFU enumeration. From these data, we calculated the cellular LOD using the method described in reference 39 implemented in R using package drc (40) for nonlinear regression. We fitted the dose-response curve with a 4-parameter logistic function (with 1/signal weighting) and determined the LOD as the concentration at which the lower bound of the 95% signal confidence interval equals the upper bound of the 95% signal confidence interval of the blank. The signal standard error of the positive samples was estimated as the pooled standard error of the lowest 3 positive samples. With this method, we determined the bounds of our limit of detection to be in the low hundreds of M. tuberculosis CFU. The overall RLU signals across the cell concentration range were higher for the 72-hour total assay time condition, and we note that, despite an additional 48 hours in plate at 37°C, the cell numbers per well are not dramatically different between the 24- and 72-hour conditions, corresponding to less than 1 cell division for cells in well, indicating the importance of metabolic adaptation to enhance signal output after TM4-nluc phage infection (see Discussion).

### Seventy-two-hour drug susceptibility testing of auxotroph and virulent M. tuberculosis strains with a panel of diverse antibiotics.

**FIG 3:**
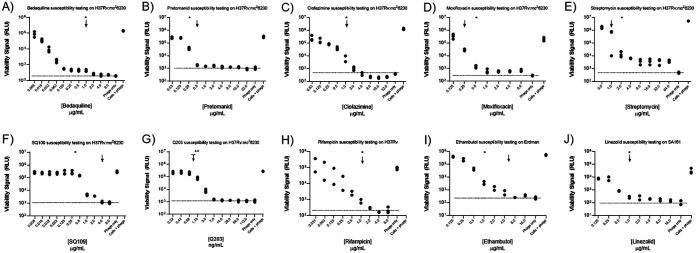
Seventy-two-hour drug susceptibility testing of auxotroph and virulent M. tuberculosis strains with a panel of diverse antibiotics. Following a preculture period in rich medium without antibiotic, M. tuberculosis cells were washed and resuspended in detergent-free rich medium with a 2-fold dilution series of antibiotic concentrations. After 48 hours of antibiotic exposure, phage was added to wells for a 24-hour infection/nanoluciferase production period. Results are displayed with viability signal (RLU) on the *y* axis and the antibiotic concentration assayed (in ascending order) on the *x* axis. For both the M. tuberculosis auxotroph H37Rv mc^2^6230 (A to G) and virulent clinical M. tuberculosis strains H37Rv (H), Erdman (I), and SA161 (J), there is ≥99% inhibition of nanoluciferase light production compared to control wells without antibiotics (indicated by asterisks) within 1 dilution of the antibiotic critical concentration (indicated by arrows). The exceptions to this are for ethambutol treatment of the Erdman strain, which resulted in ≥99% inhibition two dilutions below the critical concentration and, for novel compounds SQ109 and Q203, treatment of H37Rv mc^2^6230. We observed 99% inhibition of light production following SQ109 treatment only at concentrations 10 times the reported MIC. In the instance of Q203, we benchmarked against the MIC_50_ (capped arrow) and confirmed this value (double asterisks). Dashed lines indicate the noise floor as determined by phage-only background RLU. Antibiotics tested include bedaquiline (A), pretomanid (B), clofazimine (C), moxifloxacin (D), streptomycin (E), SQ109 (F), Q203 (G), rifampin (H), ethambutol (I), and linezolid (J). In all cases, cells plus phage represents the RLU values from infected cells in the absence of antibiotic exposure.

With TM4-nluc and this experimental system to interrogate M. tuberculosis cell health, we next looked at this system for viability testing in the context of antibiotic treatment. As an extension of the LOD results described above, in order to maximize signal/noise over our phage-only background for these experiments, we targeted an inoculation of high 10^5^ to 10^6^ M. tuberculosis CFU/well in a 96-well plate. We followed a protocol most closely resembling the 72-hour assay from above and used the pansusceptible M. tuberculosis strain H37Rv mc^2^6230 (auxotroph) (Fig. 3A to G) and wild-type virulent M. tuberculosis strains (Fig. 3H to J) from the Euro-American lineage (H37Rv [Fig. 3H] and Erdman [Fig. 3I]) and a representative of the East Asian-Beijing lineage (SA161 [Fig. 3J]). In these experiments, we prepared 2-fold serial dilution series of antibiotics to flank the WHO-reported critical concentration for each respective drug or the reported MICs for test compounds SQ109 and Q203 (Fig. 3, arrows) (16, 41, 42). After preculture, cells were exposed to antibiotic for 48 hours followed by the addition of TM4-nluc phage and overnight incubation. Accordingly, using this method, cells were exposed to fixed antibiotic concentrations for 48 hours, followed by a 5% reduction of drug exposure concentration for 24 hours (resulting from phage addition [see Materials and Methods]).

For both the H37Rv mc^2^6230 auxotroph (Fig. 3A to G) and virulent M. tuberculosis strains (Fig. 3H to J), we observed inhibition of Nluc production in a dose-response correlated with antibiotic exposure. As a measure of viability and antibiotic susceptibility, RLU production decreased as antibiotic concentration increased, and we observed ≥99% inhibition of RLU signal below or within one dilution of the critical concentration for each antibiotic tested (Fig. 3, asterisks). The lone exception to this pattern was observed with H37Rv mc^2^6230 exposed to SQ109, for which 99% inhibition of RLU signal was observed at ∼10 times the reported MIC (43). At the lowest drug treatment levels, the measured RLU approximated those of the cells plus phage wells (without antibiotics), whereas at supra-MIC antibiotic concentrations, the luminescence approximated that of the phage-only negative-control wells. These data suggest that using this system as a measure of antibiotic susceptibility can be achieved for antibiotics exerting diverse mechanisms of action. In keeping with the concept that phage-mediated viability tests are proxies for metabolic homeostasis, we anticipated that reduction of ATP content through treatment with the ATP synthase inhibitor, bedaquiline (Fig. 3A), or electron transport chain targeting, Q203 (Fig. 3G), would have profound consequences for the production of the TM4-delivered DNA construct. Similarly, the performance of this system as a readout for the activity of antibiotics that inhibit core processes of DNA transcription (rifampin [Fig. 3H]) and RNA translation (streptomycin [Fig. 3E] and linezolid [Fig. 3J]) is anticipated. Under the experimental conditions used here, pretomanid (Fig. 3B) and ethambutol (Fig. 3I) are cell wall-active antibiotics rather than inhibitors of “central dogma” functions, and the decreased production of the Nluc reporter suggests at least a 2-step readout of sensitivity beginning with disruption of structural physiology of the cell and subsequent perturbation to transcription-translation machinery. The mechanism-agnostic nature of the phage-mediated approach is demonstrated further from the results interrogating the impact of antibiotics that inhibit DNA replication (moxifloxacin [Fig. 3D]) and antibiotics with uncertain (clofazimine [Fig. 3C]) or multiple (SQ109 [Fig. 3F]) mechanisms of action.

### Drug susceptibility testing with drug-resistant M. tuberculosis auxotrophs: isoniazid and MDR.

Results reported above assess TM4-nluc performance in the cellular limit of detection and in the context of susceptibility testing for a range of antibiotics against drug-susceptible auxotrophic and virulent clinical M. tuberculosis strains. To understand performance of the TM4-nluc system in drug resistance testing, we used two different drug-resistant M. tuberculosis auxotrophic strains: an isoniazid-monoresistant strain (H37Rv mc^2^8243; *katG* W728stop) and an isoniazid- and rifampin-resistant MDR strain (H37Rv mc^2^8251; *katG* S315N, *rpoB* H445Y) (35).

**FIG 4:**
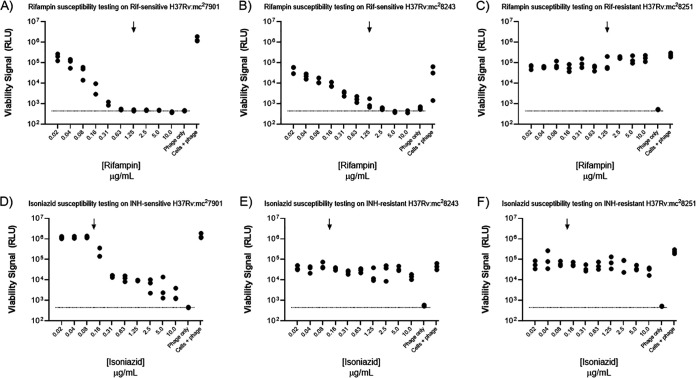
Using defined drug-sensitive and -resistant M. tuberculosis auxotrophs TM4-nluc enables 72-hour resistance testing of rifampin and isoniazid. Following a preculture period in rich medium without antibiotic, M. tuberculosis cells were washed and resuspended in detergent-free rich medium with a 10-step, 2-fold dilution series of antibiotics, rifampin (A to C) or isoniazid (D to F). The pan-susceptible isogenic parent strain, H37Rv mc^2^7901 (A to C), demonstrates a dose-dependent inhibition of nanoluciferase light production for both rifampin and isoniazid at or below the critical concentration (indicated by an arrow). The rifampin-sensitive/isoniazid monoresistant strain H37Rv mc^2^8243 (*katG* W728stop) demonstrates a dose-dependent inhibition of nanoluciferase light production upon exposure to rifampin (B) but light production approximating the untreated control in the isoniazid series (E). In the isoniazid- and rifampin-resistant MDR strain H37Rv mc^2^8251 (*katG* S315N *rpoB* H445Y), there is no reduction in nanoluciferase signal generated regardless of the antibiotic, or treatment concentration, up to 10 times the critical concentration for rifampin (C) and 100 times the critical concentration for isoniazid (F). Results are displayed with viability signal (RLU) on the *y* axis and the antibiotic concentration assayed (in ascending order) on the *x* axis. Dashed lines indicate the noise floor as determined by phage-only background RLU. In all cases, cells plus phage represents the RLU values from infected cells in the absence of antibiotic exposure.

For both rifampin (Fig. 4A to C) and isoniazid (Fig. 4D to F), there is a dose-dependent inhibition of Nluc production and luminescent signal generation in the drug-sensitive isogenic control strain (H37Rv mc^2^7901). For rifampin treatment of the rifampin-resistant auxotroph H37Rv mc^2^8251, we were unable to detect any decrease in light production, with drug concentrations in excess of 10-fold the rifampin MIC. Similarly, for two genotypically distinct isoniazid-resistant strains, isoniazid exposure failed to result in a decrease in Nluc/luminescence production. In aggregate, these results indicate that the TM4-nluc reagent and experimental system described here are capable of identifying drug susceptibility and drug resistance for two frontline antibiotics, rifampin and isoniazid. In addition, these results support the use of TM4-nluc in identifying antibiotic monoresistance, as well as combinatorial resistance, accurately identifying an M. tuberculosis auxotroph strain with a defined MDR genotype/phenotype compared to the isogenic drug-sensitive parent strain.

## DISCUSSION

This work describes the development of an improved reporter mycobacteriophage, TM4-nluc, and its validation for detection and phenotypic DST of M. tuberculosis. This reporter phage demonstrates high sensitivity and a cellular limit of detection of ≤10^2^ M. tuberculosis CFU. By interrogating TM4-nluc performance in DST assays with multiple drug-sensitive and -resistant strains of M. tuberculosis (auxotroph and virulent), we demonstrate that a preculture followed by a standardized 72-hour drug exposure-to-result period generates accurate DST results for multiple first- and second-line antitubercular antibiotics with varied mechanisms of action, including all components of the BPaL regimen.

In this work, all experiments were conducted with M. tuberculosis isolates in an *in vitro* preculture and culture environment. We observed that a critical parameter to achieve maximum cellular sensitivity is metabolic homeostasis. This was demonstrated in experiments determining the LOD, with brighter signal and improved assay sensitivity when comparing results from 72-hour experiments versus 24 hours (Fig. 2). In both cases, the total infection time was the same; however, the 72-hour experiment included an adaptation period of 48 hours, whereas the shorter duration experiment was designed with phage infection proceeding immediately after cells were placed in fresh (detergent-free) growth medium. While there may be alternate explanations, we considered likely causes to be (i) increased incubation time allowing for additional replications in the 72-hour condition with a commensurate increase in signal due to increased cell number, (ii) increased acclimation time in detergent-free medium allowing for a longer recovery/M. tuberculosis cell wall restructuring to a more phage-permissive state, or (iii) increased acclimation time in detergent-free medium allowing for metabolic recovery under altered growth conditions and therefore resulting in more robust expression of the introduced reporter cassette. Despite an additional 48 hours in the plate at 37°C, the cell numbers per well were not dramatically different between the 24- and 72-hour conditions, corresponding to less than 1 cell division. From our data, we cannot definitively distinguish between cell wall restructuring and metabolic recovery; however, if we consider these to be variations on the theme of homeostasis, then our results reinforce importance of cell health as a critical determinant underlying phage-mediated viability monitoring.

Expanding on “metabolic homeostasis” within a population of genotypically identical organisms, the concept of M. tuberculosis phenotypic heterogeneity has received substantial attention through studies monitoring responses of single cells or microcolonies (44–47), and previous work with isoniazid susceptibility testing using the green fluorescent protein (GFP) fluorophage ϕ^2^GFP10 has demonstrated that mycobacteriophages are capable of distinguishing variable phenotypic drug susceptibilities of individual cells within a genotypically uniform larger population (31). In the case of the TM4-nluc described here, the reporter signal output is a reflection of the entire population of cells assayed, rather than the phenotypic state of a single cell. We observed that the luminescence signal from cells treated with the highest concentrations of drug was elevated compared to the phage-only background (streptomycin [Fig. 3E]; isoniazid [Fig. 4D]), and we speculate that these signals were possibly derived from a minority population that maintained metabolic activity in spite of exposure to high concentrations of antibiotics (31, 48). Accordingly, testing an antibiotic phenotypic response in a stable rich environment enables differentiation of strains with subtly different susceptibility responses. This raises the possibility of using phages bearing different reporter cassettes to explore different aspects of M. tuberculosis response to perturbation, ranging from basic/foundational research with single-cell monitoring using fluorophages to deriving rapid viability data from populations of cells with luminescent phages like TM4-nluc, described here.

Considering drug susceptibility testing, particularly for antibiotics with diverse, or ill-defined, mechanisms of action and resistance mutations, the results reported here extend on previous work that has demonstrated the ability of TM4 to provide readouts on cellular responses to antibiotic exposure (25–34, 49). With TM4-nluc, we observed that there is an antibiotic dose-dependent inhibition of luciferase signal for all test antibiotics interrogated here (rifampin, isoniazid, ethambutol, moxifloxacin, clofazimine, bedaquiline, pretomanid, linezolid, SQ109, and Q203) (Fig. 3 and 4). While this is not a comprehensive list of antitubercular antibiotics, the diversity of mechanisms of action that are compatible with phage-mediated viability monitoring suggest that this approach could be valuable in the context of research and development for novel compound screening or as a comparatively rapid phenotypic DST reference complement to genotypic methods. In support of this, for all compounds tested on antibiotic-susceptible strains, we observed a dose-response inhibition of Nluc production after TM4-nluc infection. With the exception of SQ109, for all compounds, there was a ≥99% inhibition of signal below or within one drug dilution of the MIC, including all components of the BPaL regimen (Fig. 3). Testing SQ109, we observed a concentration-dependent dose-response pattern to Nluc RLU production; however, drug concentrations of approximately 10 times the reported MIC were required to achieve 99% inhibition. We hypothesize that there is a slow perturbation of M. tuberculosis metabolism when exposed to SQ109, and this may represent a case in which additional assay optimization is required to align growth- versus phage-based MIC values. The observation that there is a dose-response curve in the phage assay suggests that correlations between clinical cutoffs and *in vitro* assays can be identified. As a critical extension to drug susceptibility testing, we used TM4-nluc for identification of strains with drug resistance to both rifampin (monoresistant) (Fig. 4A to C) or rifampin and isoniazid/MDR resistance (Fig. 4D to F). We did not assess other combinations of drug resistance genotypes/phenotypes, but our combined results lead us to hypothesize that TM4-nluc can be used to identify M. tuberculosis strains with combinatorial susceptibility/resistance profiles, ranging across antibiotic classes and resistance phenotypes.

These data support the use of TM4-nluc in the context of interrogating M. tuberculosis viability/metabolic homeostasis in response to different antibiotic treatments or compound screens and potentially as a tool for characterizing genotype-phenotype relationships of wild-type and mutant M. tuberculosis strains in response to different environments or chemical treatments. Here, all experiments were conducted on cultured M. tuberculosis; however, previous studies with ffLuc or green fluorescent protein (ϕ^2^GFP10) reporter phages demonstrated that TM4-based reporter phages are compatible with clinical/sputum-derived samples after disinfection with sodium hydroxide (NaOH) (29, 31, 32). Those reports found that NaOH treatment removes mycobacteriophage receptors, thus necessitating culturing of M. tuberculosis prior to phage analysis. The enhanced activity of the TM4-nluc system (brighter signal, improved cellular limit of detection) should shorten the time to detection over previous studies on clinical samples. This, in combination with the incorporation of *p*-nitro-α-acetylamino-β-hydroxy propiophenone to distinguish between M. tuberculosis and nontuberculous mycobacteria (26), should allow for the application of this system on clinical samples. With additional validation on expanded libraries of both drug-sensitive and -resistant clinical strains of M. tuberculosis, we anticipate the value of phage-mediated quantitative DST as a complement to molecular/genotypic DST in clinical reference settings. In this regard, the potential diagnostic capacity of most phenotypic and molecular DST methods is underutilized, as they are used in a binary, or one-size-fits-all, approach. In the absence of routine quantitative DST, M. tuberculosis strains are simply identified only as susceptible or resistant without determination of the level of drug resistance, the presence and level of antibiotic cross-resistance, and the impact of associated achievable drug concentration. This may lead to unnecessary discontinuation of a key drug or incorrect diagnosis of an individual as an MDR-TB or XDR-TB patient. Therefore, sensitive quantitative phenotypic DST approaches such as the system described here can play a critical role in validating the clinical significance of a specific mutation in a particular patient or in identifying new mutations with unknown phenotypic and clinical impact. The genotype-agnostic nature of the readout, the breadth of compatible antibiotics/mechanisms of action demonstrated already, and the potential to interrogate phenotypic responses under a range of environmental conditions all suggest value in more widespread use of TM4-nluc.

## MATERIALS AND METHODS

### Culturing methods.

All the mycobacterial strains used in this study were obtained from laboratory stocks and are listed in Table 1. Mycobacterial cells were resuscitated from glycerol stock in fully supplemented 7H9 media to an optical density at 600 nm (OD_600_) of 1.0 and used as a seed culture to subculture for the next 3 weeks before going back to the glycerol stocks. Routinely, 10 ml of mycobacterial cultures was grown in 50-ml conical vials by incubating on a Cel-Gro tissue culture rotator wheel (Thermo Scientific) at 37°C. Middlebrook 7H9 supplemented with 0.5% glycerol (vol/vol), 0.10% Tween 80, and 10% albumin-dextrose-catalase (ADC; BD Biosciences) was used a liquid culture medium. 7H10 agar supplemented with 10% oleic acid-albumin-dextrose-catalase (OADC; BD Biosciences), 0.5% glycerol (vol/vol), and 0.10% Tween 80 was used as a solid culture medium. Wherever needed, nutrient supplements were used at the following concentrations: 24 μg/ml of calcium pantothenate, 50 μg/ml of methionine, and 50 μg/ml leucine. All the chemicals, unless specified, were obtained from Sigma-Aldrich or Thermo (Fisher) Scientific.

**TABLE 1:** Strains used in this study

Strain	Species	Genotype	Drug resistance^*a*^	Reference no.
mc²6230	*M. tuberculosis*	Δ*panCD* Δ*RD1*		31, 61
mc²6206	*M. tuberculosis*	Δ*leuCD* Δ*panCD*		57
mc²7901	*M. tuberculosis*	*ΔleuCD ΔpanCD ΔmetA*		35
mc²8243	*M. tuberculosis*	*ΔleuCD ΔpanCD ΔmetA katG* W728stop	INH	35
mc²8251	*M. tuberculosis*	Δ*leuCD* Δ*panCD* Δ*metA rpoB* H445Y *katG* S315N	INH, RIF	35
mc²155	*M. smegmatis*	EptB1 stop(Ochre)241L EptC1 K3stop(Ochre)		58, 62

a INH, isoniazid; RIF, rifampin.

### Generation of TM4-nluc phage.

M. tuberculosis codon-optimized nanoluciferase (nluc_M. tuberculosis_) was synthesized by GenScript Biotechnology and cloned into pMV261-derived plasmid (50, 51) using EcoRV/PsiI restriction sites. The resulting plasmid, pYUB2659, expresses Nluc from the pL promoter of bacteriophage L5 (52) and has the following features: a unique PacI site (53), pAL5000 origin of replication (54), 600-bp region flanking D29 cohesive ends (cos site) (55), and lambda cos site (56). The plasmid pYUB2659 was introduced into the TM4-derived phage backbone phAE159 using the standard *in vitro* packaging protocol (57) and electroporated into M. smegmatis mc^2^155 (57, 58) to obtain TM4-nluc phage plaques (Fig. 1). Individual plaques were picked and propagated at 30°C to obtain high-titer TM4-nluc phage (57). Phage titers were determined by spotting 5 μl of 10-fold serial dilution on M. smegmatis mc^2^155 lawns (57). Nluc activity was determined in high-titer phage lysates using the recommended standard procedure (described in “Nanoluciferase activity assay” below).

### Removal/separation of background nanoluciferase protein from phage lysates.

Two methods were optimized for reduction of Nluc protein present as a by-product in the preparation of TM4-nluc phage lysates: (i) anion-exchange chromatography (AEX) followed by hydrophobic interaction chromatography (HIC) and (ii) ultracentrifugation. Chromatography was performed at ambient temperature, and all centrifugation was performed at 4°C.

### (i) AEX.

TM4-nluc phage (>10^9^ PFU/ml) was mixed 1:1 with AEX-A loading buffer (to a final concentration of 10 mM MgCl_2_, 2 mM CaCl_2_, 50 mM Tris-Cl [pH 6.7], and 0.05% Tween 80) and loaded, at 3 ml/min, on a disposable NatriFlo HD-Q membrane adsorber (Millipore) column preequilibrated with AEX-A buffer. The column was washed with a 10-bed volume of AEX-A buffer followed by a 100-bed-volume wash with AEX-A buffer containing 250 mM NaCl (AEX-B). Bound phages were eluted by a step gradient at 1 M NaCl in AEX-A buffer (AEX-C) and collected in 1-ml fractions. Purified fractions with the highest phage titer/Nluc (titer and Nluc activity determined as described) activity ratio were pooled and used for HIC.

### (ii) HIC.

Ammonium sulfate concentration was increased to 700 mM in the pooled fraction by dropwise addition of (NH_4_)_2_SO_4_-saturated AEX-A buffer with constant stirring (59). The sample was loaded on a HiTrap butyl FF column at 1 ml/min and on a column preequilibrated with HIC-A [AEX-A with 700 mM (NH_4_)_2_SO_4_], followed by a wash with a 10-bed volume of the same. Bound phages were eluted by a 36-bed-volume linear gradient of 700-0 mM (NH_4_)_2_SO_4_ in AEX-A buffer followed by a final 12-bed-volume elution with AEX-A. One-milliliter fractions were collected and pooled into two groups, based on phage titer/Nluc activity ratio.

### (iii) Buffer exchange.

Pooled fractions were centrifuged at 20,000 × *g* for 3 hours at 6°C in high-*g* polypropylene centrifuge tubes. The supernatant was gently removed, and the phage pellet was resuspended in mycobacteriophage (MP) buffer overnight without shaking at 4°C. Phage titer/Nluc activity was determined prior to subsequent use.

### (iv) TM4-nluc purification by ultracentrifugation.

Briefly, cesium chloride (CsCl) solution (density, 1.54 g/ml) was prepared by dissolving 100 g of molecular biology-grade cesium chloride (Merck Millipore, USA; catalog no. 219650) in 100 ml of water and filtered through a 0.22-μM membrane filter. We transferred 25 ml of this solution to 38.5-ml ultraclear tubes (Beckman Coulter, USA; catalog no. 344058), and 13 ml of TM4-nluc lysate in MP buffer (prepared as described above) was layered slowly above the CsCl solution. Tubes were centrifuged for 22 h at 28,000 rpm (96,281 × *g*) at 4°C in a swinging-bucket rotor (SW 32 Ti) (Beckman Coulter, USA). Following centrifugation, tubes contained three visible fractions, which were individually collected starting from the top layer by means of puncturing with an 18-gauge needle. RLU and titers of fractions were measured as described above. The top and bottom layer fractions were low yield and discarded. The middle layer fraction had the optimal RLU/titer ratio and was used in further assays.

### LOD and phenotypic DST assays.

From glycerol stock, M. tuberculosis cells were inoculated to a target OD_600_ of ∼0.1 in 10 ml fully supplemented 7H9 medium containing Tween 80. Cells were incubated for 72 hours at 37°C with agitation. Following this preculture period, the resultant M. tuberculosis cells were washed twice with detergent-free (fully supplemented 7H9 without Tween 80) medium and resuspended in the same medium to a final OD_600_ of 0.1 to 0.2. Cells were incubated as standing culture for 24 hours at 37°C prior to LOD and DST assay setup.

### (i) LOD.

We aliquoted 90 μl of 10-fold serially diluted cells in four sets of triplicates in 96-well flat-bottom plates. Two sets were used for each 24- and 72-hour LOD determination. For the 24-hour LOD, 10 μl of phage was immediately added to one set, and the other set of 10-fold serially diluted cells was plated for CFU enumeration on 7H10 agar plates. For the 72-hour LOD, the plates were incubated for an additional 48 hours at 37°C. Subsequently, 10 μl of phage was added to one set, and the other set was plated on 7H10 agar plates for CFU enumeration. The final concentration of phage in each well was 3.5 × 10^7^ PFU/ml. In each case, the plates were incubated at 37°C for 24 additional hours after phage addition, and the nanoluciferase activity was determined using the manufacturer-recommended standard procedure (described in “Nanoluciferase activity assay” below). The number of M. tuberculosis cells in each well at the time of phage infection was enumerated by counting for colonies after 3 weeks of incubation at 37°C in a sealed plastic bag.

### (ii) DST.

Ninety microliters of 2-fold serially diluted drug dilutions was aliquoted in a set of triplicates along with no-drug control triplicate wells. We added 5 μl of M. tuberculosis cells with an OD_600_ of 0.1 to 0.2 to 90 μl of drug dilutions and the control wells. The plates were incubated at 37°C for 48 hours before adding 5 μl of phage to each treatment and phage-only control well. The final concentration of phage in each well was 2 × 10^7^ PFU/ml. Plates were incubated at 37°C for 24 additional hours after phage addition, and the nanoluciferase activity was determined using the recommended standard procedure (described in “Nanoluciferase activity assay” below).

### Nanoluciferase activity assay.

Nanoluciferase activity was determined as recommended in the Nano-Glo luciferase assay system manual. Briefly, Nano-Glo substrate was diluted 1:50 in Nano-Glo buffer and mixed 1:1 (vol/vol) with samples and read on a BioTek Synergy H1 plate reader using the following settings: luminescent fiber, 135 gain, 0.1 s read time, and 1 mm read height. The method and settings were consistent for determination of RLU in M. smegmatis/phage propagation, assessment of phage cleanup during purification, and in experiments with auxotroph and virulent M. tuberculosis, with the exception that for M. tuberculosis experiments, after addition of substrate, plates were covered with adhesive, optically clear plate sealer (Microseal B PCR plate-sealing film, adhesive, optical no. MSB1001; Bio-Rad) and decontaminated, and luminescence was read immediately.

## Supplementary Material

Supplemental file 1
