# Supplementary material for: Nanoluciferase Reporter Mycobacteriophage for Sensitive and Rapid Detection of Mycobacterium tuberculosis Drug Susceptibility
Source: J Bacteriol. 2020 Oct 22;202(22):e00411-20. doi: 10.1128/JB.00411-20 (PMC7585058; doi:10.1128/JB.00411-20)
Supplement: Supplemental file 1 [file JB.00411-20-s0001.pdf]

A)

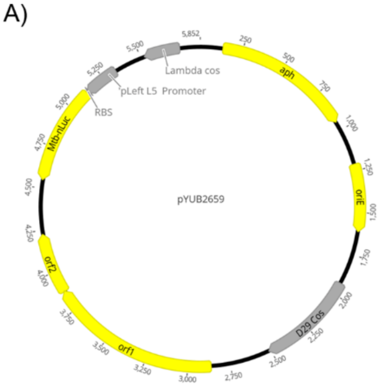

B)

```
>nluc_mtb
atg gtg ttc acc ctg gag gac ttc gtg ggc gac tgg cgc cag acc gcc ggc tac aac ctg
 M   V   F   T   L   E   D   F   V   G   D   W   R   Q   T   A   G   Y   N   L
gac cag gtg ctg gag cag ggc ggc gtg tcg tcg ctg ttc cag aac ctg ggc gtg tcg gtg
 D   Q   V   L   E   Q   G   G   V   S   S   L   F   Q   N   L   G   V   S   V
acc ccg atc cag cgc atc gtg ctg tcg ggc gag aac ggc ctg aag atc gac atc cac gtg
 T   P   I   Q   R   I   V   L   S   G   E   N   G   L   K   I   D   I   H   V
atc atc ccg tac gag ggc ctg tcg ggc gac cag atg ggc cag atc gag aag atc ttc aag
 I   I   P   Y   E   G   L   S   G   D   Q   M   G   Q   I   E   K   I   F   K
gtg gtg tac ccg gtg gac gac cac cac ttc aag gtg atc ctg cac tac ggc acc ctg gtg
 V   V   Y   P   V   D   D   H   H   F   K   V   I   L   H   Y   G   T   L   V
atc gac ggc gtg acc ccg aac atg atc gac tac ttc ggc cgc ccg tac gag ggc atc gcc
 I   D   G   V   T   P   N   M   I   D   Y   F   G   R   P   Y   E   G   I   A
gtg ttc gac ggc aag aag atc acc gtg acc ggc acc ctg tgg aac ggc aac aag atc atc
 V   F   D   G   K   K   I   T   V   T   G   T   L   W   N   G   N   K   I   I
gac gag cgc ctg atc aac ccg gac ggc tcg ctg ctg ttc cgc gtg acc atc aac ggc gtg
 D   E   R   L   I   N   P   D   G   S   L   L   F   R   V   T   I   N   G   V
acc ggc tgg cgc ctg tgc gag cgc atc ctg gcc gga tcc ccg gcg aga gct tcc cac cac
 T   G   W   R   L   C   E   R   I   L   A   G   S   P   A   R   A   S   H   H
cac cac cac cac cac cac taa
 H   H   H   H   H   H   -
```

Figure S1 *– A) Vector map and nluc$_{MTB}$ sequence. Vector map of pYUB2659 expressing codon optimized NanoLuciferase (nLuc$_{MTB}$) and was used for the construction of TM4-nluc . B) Nucleotide sequence of the nluc$_{MTB}$ (shown in bold) and the corresponding amino acid sequence (shown below). Eight histidine tag was added at the C-terminal separated by a flexible linker (shown in green). The vector pNL1.1[Nluc] was used as a source for the Nanoluciferase sequence (https://www.ncbi.nlm.nih.gov/nuccore/JQ437370).*

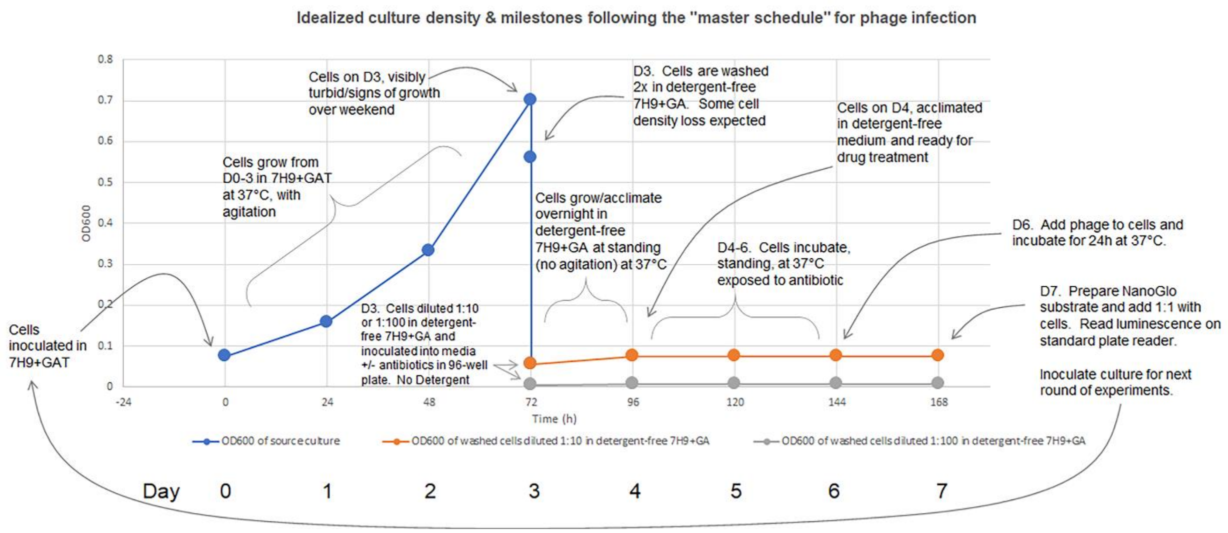

Figure S2 – Annotated workflow of TM4-nluc-mediated DST and viability monitoring. *Idealized growth curves are shown with OD_{600} on the Y-axis, and time (in hours) on the X-axis. This schematic depicts a 7-day total turn-around time ("Friday-to-Friday," as described here), with critical manipulations and incubations annotated. Following inoculation at T0 h, MTB cells are pre-cultured in rich, detergent-containing, medium and monitored for growth for 72 h, after which they are washed and transitioned to detergent-free rich medium for 24 h. Antibiotic treatment is initiated at T96 h and proceeds for 48 h to T144 h, at which point phage is added to the wells for a 24 h infection/incubation period. NanoGlo substrate is added to plates and luminescence signal is read.*

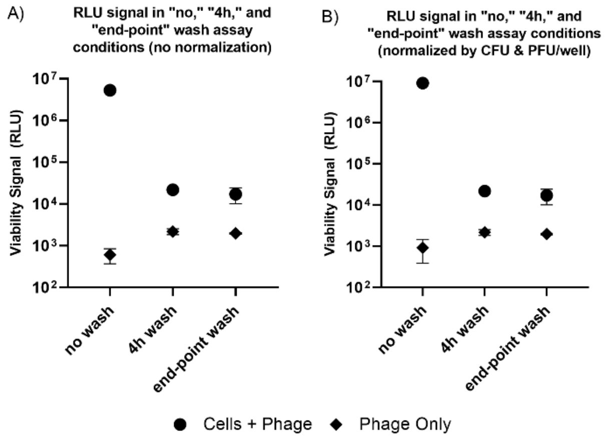

***Figure S3 – Impact of vacuum filtration on Nluc signal:noise**. RLU from "phage only" background and "cells + phage" signal comparing assay designs of "no wash" versus vacuum filtration washing in 0.2 µm 96 well PVDF filter plate after 4 hours of phage infection ("4h wash") or immediately prior to adding substrate and reading RLU ("end-point wash"). A) "No wash" assays demonstrate superior signal compared to either wash condition when considering raw RLU data, and B) this persists after normalizing for CFU and PFU inputs across conditions.*
